# Knowledge and factors influencing long-acting reversible contraceptives use among women of reproductive age in Nigeria

**DOI:** 10.12688/gatesopenres.12902.3

**Published:** 2020-05-20

**Authors:** Obasanjo Afolabi Bolarinwa, Olalekan Seun Olagunju

**Affiliations:** 1 Department of Demography and Social Statistics, Obafemi Awolowo University, Ile-Ife, Osun State, 1000009, Nigeria

**Keywords:** Knowledge, Factors, LARC, Contraceptive use, Women, Reproductive age, Influencing, Nigeria.

## Abstract

**Background:** Approximately 48% of unintended pregnancies occur as a result of contraceptives failure around the world, which is mostly due to incorrect use, poor adherence, and/or technology failure. Long-acting reversible contraceptive (LARC) methods have been developed to close this gap. The main aim of this study is to identify factors associated with the use of LARCs among women of reproductive age and to examine the relationship between knowledge of LARCs and the current use of LARCs in Nigeria.

**Methods: **This study assessed the PMA2020 methodology and secondary dataset using female datasets from PMA 2016 (Round 3) exercise. The survey was conducted out in seven states of Nigeria. The target population for this study was women of reproductive age (15-49 years) currently using contraception prior to the survey. The sample size of women that met the inclusion criteria was 1927. The data were analyzed using frequency distribution, chi-square, and logistic regression at a 5% level of significance.

**Results: **The results showed that 21.0% of women were using traditional methods. 14.8% of the sampled women were using LARCs methods. Findings further showed that at both levels of analyses, there is a significant relationship (P<0.05 and P=0.00 for binary and logistic regression, respectively) between knowledge of LARCs and the use of LARCs. This means that knowledge of LARCs and socio-demographic variables among women of reproductive age in Nigeria can influence the use of LARCs.

**Conclusions: **We concluded in this study that 14.8% of women using contraception were using LARCs. Additionally, the level of education, age of women, household wealth, and the number of living children were significantly associated with using LARCs in Nigeria. Also, when discussing contraception with women, health care practitioners should discuss the risks and benefits of LARCs with women of reproductive age and recommend them as a first-line method.

## Introduction

The rising use of contraception in Nigeria has given women the ability to choose the number and spacing of their children. It has also presented them with various remarkable life-saving benefits, such as the reduction in maternal and infant mortality, proper child spacing, and better postpartum health outcomes. Recently, the expansion in choice of contraceptives available has given women the option of adopting the use of Long-acting reversible contraceptives (LARCs), which are implant and intrauterine device contraceptive methods that are highly effective and convenient with an added advantage of being long-lasting, require little or no maintenance. It has much better compliance rates than other hormonal methods and is also cost-effective. LARCs are ideal pregnancy prevention options for many women compared with shorter-term and user-dependent methods, both of which increase the risk of non-compliance related method failure ^1– 4^.

Long-acting devices, when initiated, provide at least three years of continuous pregnancy protection for women, and can give up to 10 years of protection. These devices are 99% effective because they are not subject to errors in use, unlike short-acting methods ^2^. Also, LARC methods can reduce the gap between "typical use" and perfect use" failure rates ^1^. Approximately 48% of unintended pregnancies occur as a result of contraceptives failure around the world ^5^, which is mostly due to incorrect use, poor adherence, and/or technology failure. This can be avoided with the use of LARC methods, because they are not dependent on compliance with a pill-taking regimen, remembering to change a patch or ring, or arranging an appointment with physicians ^6, 7^.

Several studies have established that women in sub-Saharan Africa are often unable to obtain or use modern contraception, particularly the long-acting methods, for many reasons associated with both supply and demand-side ^8, 9^. Nigeria is not particularly exempted from Long-Acting Contraceptives Prevalent use of 3.1% ^10^.

'Nigeria's total fertility rate (5.5) is one of the lowest in sub-Saharan Africa and globally. This is primarily due to her high unmet need for family planning of (21.8%). The use of contraception is relatively low (17.1%), and this also reflected in the number of women that subscribed to LARCs despite being the most cost-effective contraceptives. In Nigeria, knowledge about LARCs in terms of an intrauterine device (IUD) and implant shows that 36.8% of women have knowledge of IUD and 49.5% of implants ^11, 12^, which is also low.

Despite the efficacy and safety of LARCs, the use is not widespread among women of reproductive age in Nigeria. Hence, this paper examined the relationship between women's knowledge of LARCs and factors influencing the use of LARCs among women of reproductive age in Nigeria to guide policymakers' decisions.

This research paper sought to identify the relationship between knowledge and factors influencing the use of LARCs among women of reproductive age in Nigeria.

## Methods

### Data source

The study employed secondary data and methodology from Performance Monitoring and Accountability (PMA) 2016 dataset ^12, 13^. PMA 2016 was a cross-sectional survey carried out in 7 states of Nigeria, Anambra, Kaduna, Kano, Lagos Nasarawa, Rivers, and Taraba States between the 4th day of May to the 31st day of June 2016. The survey used aboriginal enumerators who were familiar with the enumeration areas and had a good command of the local language. A multistage sampling technique was employed, first to select enumeration areas (EAs) in each local government (LG) of the state, and to randomly select households for an interview in each selected EAs. The enumerators administered all females of reproductive age (15–49 years) living within the household chosen a female questionnaire. The information recorded on the questionnaires included the eligible 'female's background information, birth history, fertility preference, use of family planning methods, and their reproductive health information, among others. A total of 11,177 women were interviewed. The questionnaires used are available on OSF ^14^.

### Scope of study

This study was limited to the PMA2020 secondary dataset using female datasets from PMA 2016 (Round 3) exercise ^12^. It is expected to provide further insight into factors contributing to the use of long-acting contraception in Nigeria. The target population for this study was women of reproductive age (15–49) who are currently using contraception prior to the survey. Accordingly, for women who met the inclusion criteria, the sample size was 1927.

### Operational definitions and study variables

In this study, the primary outcome of interest was LARCs use among current contraceptives users (This is defined as women of reproductive age (15–49 years) that are currently using contraception or whose partner are using at the time of the survey). The study focused specifically on contraceptives users rather than all of those at risk for unintended pregnancy. The current use of a LARCs method is defined here as the use of the contraceptives implant or the IUD during the month of the interview.

Knowledge of LARCs was assessed by whether the respondent has heard of implant or IUD. Respondents were considered as having knowledge if they responded "Yes" to the question "Have you ever heard of implant or IUD" at the time of interview. A Source of information about family planning was also included in the study.

To assess women's demographic characteristics likely to influence LARCs use, selected demographic characteristics that are theoretically related to the use of LARCs were included in the analyses. These include women's level of education, household wealth index, number of births at first use of contraceptives, place of residence, age, and marital status.

To answer the stated objectives, we first present the frequency distribution of all the variables used in the study. The pattern of LARCs use was assessed by the proportion of all contraceptives users using LARCs methods by selected demographic characteristics. Knowledge of LARCs was cross-tabulated by the use of LARCs to show the relationship between the two variables, and the chi-square test was used to show this relationship. Lastly, binary logistic regression was used to estimate the odds ratio adjusting for demographic factors influencing the use of LARCs.

### Data processing and analysis

Data was exported to Stata version 14 for analysis. Descriptive statistics, including frequencies and proportions, were used to summarize the variables. Binary logistic regression was used. Adjusted odds ratio (AOR) with a 95% confidence interval were estimated to show the strengths of associations. Finally, a p-value of less than 0.05 in the multivariable logistic regression analysis was used to identify variables significantly associated with long-acting and reversible family planning method utilization.

## Results

Table 1 shows the distribution of respondents that are currently using contraception by selected socio-demographic characteristics. A total of 1,927 females were found to be currently using contraception in the study. The mean age of respondents was 31.3 years, and more than 40% of the current users fell within the age range of 25–34 years. Concerning the level of education, almost half (49.7%) of females had attended secondary education and while 24.1% had higher education. Marital status shows that the majority (76.6%) of the respondents were currently married at the time of the interview. More than half (59%) of the respondents reside in urban areas, while 41% live in rural areas.

**Table 1.** Socio-demographic characteristics of respondents.

Variables	Percentage of respondents (N=1927)
Age group	
15–24	22.4
25–34	41.0
35+	36.6
**Mean (SD)**	**31.3 (7.9)**
Highest level of education	
Never attended	9.1
Primary	17.1
Secondary	49.7
Higher	24.1
Marital status	
Currently married	76.6
Divorced or separated	2.4
Widow	1.7
Never married	19.3
Place of residence	
Urban	59.0
Rural	41.0
Wealth index	
Poor	35.6
Middle	21.3
Rich	43.1
Number of children at first use of family planning	
None	31.2
1–4	52.6
5+	16.2

Regarding the wealth index, the table shows that 43.1% were from wealthy households, 35.6% from a poor household, and 21.3% were from the middle household. More than half (52.6%) of the respondents had 1–4 children before they started using contraception. Raw data are available on OSF ^14^.

**Figure 1.**
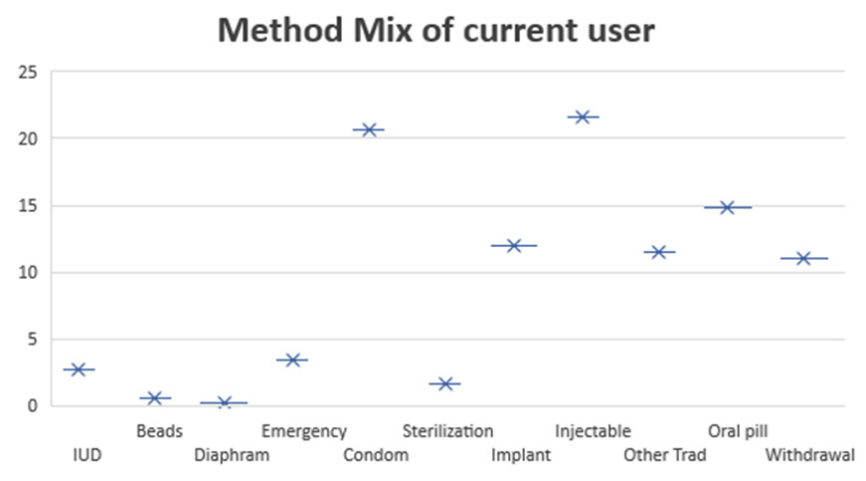
Method Mix of the current user.

The method mix of the respondents and percentage distribution is shown in Figure 1. Respondents included those who were married to or living with a man at the time of the survey and were currently using contraception.

Table 2 presents the LARCs use frequency and 95% Confidence Interval (CI) of the respondents. A total observation of 1927 was considered with 0.15 mean, 0.0081 standard error, 14.8% prevalence use of LARCs, and a confidence interval of 0.1320 - 0.1638.

**Table 2.** LARCs use frequency and 95% Confidence Interval.

Variable	Observation	Mean	Std.Err.	Prevalence of use	95% CI
LARCs use	1927	0.15	0.0081	14.8%	0.1320 – 0.1638

Table 3 presents the respondent's contraceptives awareness and knowledge of LARC methods. The table shows that 28.5% read about family planning in newspaper/magazine, 49.7% heard about it on television, and 67.2% heard on the radio. Concerning awareness at a health facility and from a health worker, 53.6% of the respondents reported that they were talked to about family planning at the health facility, and only 18.5% heard about family planning when visited by a health worker in the last 12 months. Knowledge about LARCs shows that 70.3% of women in the study had knowledge of the contraceptives implant, and 55.5% of females had knowledge of the IUD.

**Table 3.** Awareness of contraceptives methods and knowledge of long-acting contraceptive methods.

Variables	Percentage of respondents (N=1927)
Read about FP in newspaper/magazine	
No	71.5
Yes	28.5
Heard about FP on television	
No	50.3
Yes	49.7
Heard about FP on radio	
No	32.8
Yes	67.2
Talked to about FP at a health facility	
No	46.4
Yes	53.6
Visited by health worker about FP last 12 months	
No	81.5
Yes	18.5
Ever heard of implants	
No	29.7
Yes	70.3
Ever heard of intrauterine device	
No	44.5
Yes	55.5

FP, family planning.

Table 4 presents the practice of contraceptives among females who are currently using any method of contraception. The table shows that (76.5%) females that are currently using any method of contraception were using modern contraceptives (e.g., condoms, hormonal pill), and 14.8% of respondents were using LARCs.

**Table 4.** Contraceptives use.

Variables	Percentage of respondents (N=1927)
Current use of modern contraceptives method	
No	23.5
Yes	76.5
Current use of traditional contraceptives method	
No	79.0
Yes	21.0
Current use of long-acting reversible contraceptives method	
No	85.2
Yes	14.8

Table 5 presents the pattern of LARCs use among the current user of contraceptives by selected socio-demographic characteristics. The table showed that LARCs use increases as the 'respondent's reproductive age increases. More women who reside in urban areas were using LARCs compared to those in rural areas. More women with secondary education used LARC methods compared to women with no education, primary and higher education. Marital status shows that married women prefer LARCs compared to divorced/separated, widow, and never married. Concerning the household wealth index, the table shows that more women from poor households subscribed to the LARC methods compared to women from a middle and wealthy household. Lastly, the number of children at the time respondent started using contraceptives shows that more women that had 1–4 children subscribed to LARC methods compare to women with no child and women with more than four children.

**Table 5.** The Pattern of use (long-acting reversible contraceptive methods).

Variables	Percentage of respondents (N=285)
Age group	
15–24	9.8
25–34	44.6
35+	45.6
Place of residence	
Urban	51.6
Rural	48.4
Highest level of education	
Never attended	8.8
Primary	20.0
Secondary	48.4
Higher	22.8
Marital status	
Currently married	93.7
Divorced or separated	2.1
Widow	2.5
Never married	1.7
Household Wealth index	
Poor	43.5
Middle	18.3
Rich	38.2
Number of children at first use of family planning	
None	6.0
1–4	66.3
5+	27.7

Table 6 presents the association between knowledge of LARCs and the use of LARC methods among women that are currently using contraception. The table showed that at both levels of analyses (binary and multivariable logistic regression), there is a significant relationship (P<0.05 and P=0.00, respectively) between knowledge of LARCs and the use of LARCs in this study. This means that the use of LARCs can be influenced by knowledge of LARCs among women of reproductive age in Nigeria.

**Table 6.** Association between knowledge and use of long-acting reversible contraceptives (LARCs).

Variables	Use of LARCS		
Ever heard of implant	No, % (n=1,642)	Yes, % (n=285)	Total, % (N=1,927)
No	34.4	3.2	29.7
Yes	65.6	96.8	70.3
	X ^2^=113.1, P=0.000 *		
Ever heard of IUD			
No	47.0	30.2	44.5
Yes	53.0	69.8	55.5
	X ^2^=27.9, P=0.000 *		

*Significant at P < 0.05. IUD, intrauterine device.

Logistic regression was employed to assess the net effect of the selected variable theoretically related to the use of LARC methods in Table 7. The result of logistic regression showed that women who were 25 years and above, women with secondary and higher education, currently married and widow, women from rich households and women with one or more children were significantly associated with the use of LARC methods.

**Table 7.** Factors influencing the use of long-acting reversible contraceptive methods.

Variables	Odds ratio	P-value	Confidence interval
Age group (RC=15–24)			
25–34	1.67	0.05	0.9864-2.8171
35+	1.73	0.05	0.9849-3.0308
Place of residence (RC=Rural)			
Urban	0.92	0.70	0.6088-1.3919
Highest level of education (RC=Never attended)			
Primary	1.68	0.11	0.8826-3.2143
Secondary	2.64	0.00	1.4122-4.9475
Higher	3.30	0.00	1.5973-6.8293
Marital status (RC=Never married)			
Currently married	4.61	0.01	1.3566-15.6591
Divorced/separated	2.41	0.32	0.4298-13.5370
Widow	7.16	0.01	1.4163-36.2025
Household wealth (RC=Poor)			
Middle	0.88	0.61	0.5327-1.4458
Rich	0.57	0.03	0.3432-0.9442
Number of children at first use of family planning (RC=None)			
1–4	4.28	0.00	2.2500-8.1412
5+	6.08	0.00	2.9560-12.5079
Family planning discussion at a facility (RC=No)			
Yes	1.38	0.06	0.9832-1.9499
Visited by a health worker (RC=No)			
Yes	0.82	0.32	0.5617-1.2041
**Constant**	**0.00**	**0.00**	**0.0011-0.0177**

**RC, recode.**

Women who fall between the ages of 25 and 34 years were 1.67 times more likely to use LARC methods than those aged 15–24 years, and those women that 35 years and above were 1.73 times more likely to use LARC methods than those aged 15–24 years.

Level of education shows that women with secondary school education were 2.64 times more likely to use LARC methods than those that never attended school, and those women with higher education were 3.30 times more likely to use LARC methods than those that never attended school in the study. Concerning marital status, the results show that married women were 4.61 times more likely to use LARC methods than those that never married and widowed women were 7.16 times more likely to use LARC methods than those that never married.

Concerning the household wealth index, women from rich households were 0.57 times less likely to use LARC methods than women from poor households. Besides, women with 1–4 children at the time of contraceptives use were 4.28 times more likely to use LARC methods than women with no child and women with more than four children at the time of contraceptives use were 6.08 times more likely to use LARC methods than women with no child. Lastly, women who heard about family planning at a health facility were 1.38 times more likely to use LARC methods than those that heard it elsewhere.

## Discussion

This paper assessed knowledge of LARCs and factors influencing the use of LARCs among women that are currently using contraception in Nigeria. The study showed that LARCs were largely under-used among women that are currently using any contraception. To properly harness socio-economic opportunities and better child spacing, the low use of LARCs should be tackled because of its integral benefit of meeting women's reproductive needs in a context where women are redefining their reproductive lifestyle ^4, 15^.

This study shows that there was an association between 'women's knowledge of LARCs and the use of LARCs among women that are currently using contraception. This is because women's knowledge about the efficacy and safety of LARC methods may strongly influence both the selection and decision to continue to use the selected method over time. These findings were in line with previous studies that say women will opt for LARC methods as their contraceptive method of choice when they have knowledge of methods ^3, 6, 7, 16– 20^. Another study also affirmed that women's reproductive life plans are being altered as a result of misinformation, and this prompt woman to adopt methods not suitable for themselves ^21^.

Besides, the level of education was found to be associated with the use of LARCs. The possibility of women with at least secondary school education to control her reproductive need is very high. The higher the education of women, the higher the propensity that they will adopt the use of LARCs. Previous studies also corroborate this point that better-educated women have access to information on modern contraceptives, which may trigger their interest in the use of LARCs ^6, 18, 19, 22– 26^.

Women aged 25 years and above were more likely to use LARC methods as compared to women aged 15–24 years. This result is in line with previous studies, which reported that the age of mothers was found to be associated with the use of LARCs because the prevalence of LARC uses increased with age ^3, 4, 23, 24, 26^.

The number of living children at the time of contraceptives use was significantly associated with LARCs use. Suggesting that women wanted to space or limit childbirth as the number of surviving children increases. The higher the number of living children, the higher the possibility of adopting LARCs. The desire to limit the number of children will automatically come to play when women believe they have sufficient numbers of children, so rather than adopting short-lasting, long-lasting methods will be preferred ^4, 22, 23, 27^.

Furthermore, women from rich households were less likely to use LARCs. This is contrary to other studies, which found that household wealth has a positive association with the use, and wealthier women were more likely to use LARCs than poorer women ^4, 23, 24^.

Lastly, the study found that married women were more likely to use LARC methods. This is consistent with previous studies that showed that married women have good attitudes towards using LARC methods ^17, 24, 28^.

### Limitations

This study was conducted among women of reproductive age who are currently using contraception, which might not reflect a holistic view of all women of reproductive age in Nigeria. Also, the cross-sectional design used to collect the data comes with major limitations allowing us with mere hypotheses than real cause-effect relationships.

### Future suggestions

The study showed that LARCs were largely under-used among women that are currently using contraception in Nigeria. To properly harness socio-economic opportunities and better child spacing, the low use of LARCs should be tackled because of its integral benefit of meeting 'women's reproductive needs in a context where women are redefining their reproductive lifestyle. Therefore, women with lower educational levels, high wealth index, and a higher number of living children should be targeted by program strategies to control childbearing. Also, there is a need for a communication strategy that would provide correct information about LARCs’ safety and effectiveness among women of reproductive age. Lastly, when discussing contraception with women, health care practitioners should discuss the risks and benefits of LARCs with women of all ages and recommend them as a first-line method.

## Conclusions

In conclusion, findings from this study showed that 14.8% of women in Nigeria that are currently using contraception were using LARCs. Additionally, the level of education, age of women, household wealth, and several living children at the time of contraceptives use were significantly associated with the use of LARCs. Also, knowledge of LARCs was significantly associated with LARCs use. To effectively control the childbearing in Nigeria, women with lower education, high wealth index, and a high number of living children should be the target audience among the women of reproductive age in Nigeria. Also, there is a need for a communication strategy that would provide correct information about LARCs safety and effectiveness among women of reproductive age. Lastly, when discussing contraception with women, health care practitioners should discuss the risks and benefits of LARCs with women of reproductive age and recommend them as a first-line method.

## Data availability

### Underlying data

Raw data associated with this study are available on OSF. DOI: https://doi.org/10.17605/OSF.IO/C5YGV ^14^.

### Extended data

Questionnaires used in this study are available on OSF. DOI: https://doi.org/10.17605/OSF.IO/C5YGV ^14^.

Data are available under the terms of the Creative Commons Zero "No rights reserved" data waiver (CC0 1.0 Public domain dedication).
