# Supplementary material for: Knowledge and factors influencing long-acting reversible contraceptives use among women of reproductive age in Nigeria
Source: Gates Open Res. 2020 May 20;3:7. Originally published 2019 Jan 14. [Version 3] doi: 10.12688/gatesopenres.12902.3 (PMC7447856; doi:10.12688/gatesopenres.12902.3)
Supplement: Peer review history

# Peer review: Reviewer response for version 3

**DOI:** 10.21956/gatesopenres.14330.r29082

**Published:** 2020-07-30

**Authors:** Anita L. Nelson

**Affiliations:** 1 Western University of Health Sciences COMP-Northwest, Lebanon, OR, USA

This paper is a secondary analysis of the subset of data collected in 2016 which included married or cohabiting women in seven states in Nigeria who were using some method of birth control at the time. The authors sought to find associations between basic demographic data collected in the survey and use of implants and IUDs. More curiously, they also tried to determine if having heard about an IUD or an implant was associated with a higher likelihood that the woman would use one of those methods. The purpose of this work was to inform public policy to help decision makers design more targeted educational programs to increase use of implants and IUDs.

 Previous reviewers commented on the tautology represented by the primary study objective, but apparently the authors did not understand how much of a showstopper this is. It is perfectly clear that women cannot choose methods they have never heard about. No one needs to read a publication to come to that conclusion. However, the authors could review their data and provide better insights into what they characterized as “underutilization” of these methods. For example, 70.39% of women had heard of implants and 55.2% of women had heard of IUDs, but only 14.8% of women used an IUD or an implant. The vast majority of women who had heard about IUDs and implants decided NOT to use them. The more interesting research and policy question would be why did they not select one of these methods?

 Some of the answers may come from what the authors know, but did not share in the article. Do women have barriers to access to IUDs and implants? Are there financial barriers? (One might suspect not in view of the fact that it was women in lower income brackets that more frequently used these methods than the wealthier women) Are there some limitations on the sites and are all providers able to place the IUDs and implants? Are there any issues of trust with the medical system that limit women’s enthusiasm for the methods? Specifically, are they confident that doctors will remove their IUD or the implant if they have problems or want it out for other reasons?

 The authors present information about where women heard about family planning but do not use it in any of their recommendations. Radio seems to be the most common medium recalled by the women. Clearly a multi-media educational program may be helpful, but it appears radio might be a first choice for getting word out.

 Even more fundamentally, the premise of the project is that utilization rates of implants and IUDs should be higher. It is not clear why policy makers values and preferences should replace those of the woman in choosing what method she (and her partner) will use. From an ethical standpoint, it is clear that women need to know what all their options are before they can make informed choices. And all methods should be first line choices, except the ones to which the woman has medical contraindications. The authors claim throughout the paper that LARCs are largely underutilized among currently contracepting women. However, the basis for that claim “underutilized” is not substantiated, what percent to the authors think would be adequate? How was that target decided? And the authors claim that IUDs and implants are “ideal pregnancy prevention options for many women”, but clearly, they cannot be ideal if the vast majority of women decide they do not want to use those methods.

 There are many errors in terminology and extensive unnecessary duplication in the text. One example is that the authors say that Nigeria has one of the lowest fertility rates (5.5%) in Sub-Saharan Africa and globally. And they attribute that low fertility rate to high unmet need for family planning. These slip-ups can be addressed later if the authors are able to redraft this work to refocus it on the other findings they have.

 I would suggest Figure 1 be converted to a table with actual numbers. What percent of women used implants and what percent used IUDs? Which IUDs were most popular? Table 6 was extremely confusing; as I understood it, 3.2% of women who had ever heard about the implant used a LARC (presumably an IUD?) and 30.2% of women who never heard of an IUD used a LARC (presumably an implant?). I do not understand what it adds to the article.

 The discussion section only repeats findings and states if they are consistent with what other authors have reported. There is no work done to try to explain the differences – different access, different study populations, etc. There is no explanation offered of why women 15-24 years were less likely to use these methods compared to older women. The authors could try to explain that finding (are women seeking pregnancy in their early 20’s? or providers do not use in teens or nulliparous women?). Such conjecture can help the reader decide whether these findings are at all relevant to his situation.

 The real problem with this paper as it is currently drafted is that is a really local issue and may be better placed in a regional journal. Unless the authors can describe how their system works to provide to these methods, readers cannot learn lessons they can apply to their own situations. This journal is very supportive of local work, but the authors really need to consider what it is about their work that would interest other readers.

 If the authors would like to reorient the focus of their work, I would be happy to review their next version and to work with them on the English language challenges.

Is the work clearly and accurately presented and does it cite the current literature?

Partly

If applicable, is the statistical analysis and its interpretation appropriate?

No

Are all the source data underlying the results available to ensure full reproducibility?

No

Is the study design appropriate and is the work technically sound?

No

Are the conclusions drawn adequately supported by the results?

No

Are sufficient details of methods and analysis provided to allow replication by others?

No

Reviewer Expertise:

NA

I confirm that I have read this submission and believe that I have an appropriate level of expertise to confirm that it is of an acceptable scientific standard, however I have significant reservations, as outlined above.

# Peer review: Reviewer response for version 3

**DOI:** 10.21956/gatesopenres.14330.r28863

**Published:** 2020-06-01

**Authors:** Ronald Anguzu

**Affiliations:** 1 Public and Community Health Program, Institute for Health and Equity, Medical College of Wisconsin (MCW), Milwaukee, WI, USA

The authors responses are satisfactory and I approve indexing of their manuscript.

Is the work clearly and accurately presented and does it cite the current literature?

Yes

If applicable, is the statistical analysis and its interpretation appropriate?

Yes

Are all the source data underlying the results available to ensure full reproducibility?

Yes

Is the study design appropriate and is the work technically sound?

Yes

Are the conclusions drawn adequately supported by the results?

Yes

Are sufficient details of methods and analysis provided to allow replication by others?

Yes

Reviewer Expertise:

NA

I confirm that I have read this submission and believe that I have an appropriate level of expertise to confirm that it is of an acceptable scientific standard.

# Peer review: Reviewer response for version 2

**DOI:** 10.21956/gatesopenres.14313.r28811

**Published:** 2020-05-12

**Authors:** Ronald Anguzu

**Affiliations:** 1 Public and Community Health Program, Institute for Health and Equity, Medical College of Wisconsin (MCW), Milwaukee, WI, USA

This article is important in the field of prevention of unintended pregnancies as a public health problem both globally and specifically in Nigeria as the authors demonstrated. Implementation efforts to increase utility of long-acting reversible contraception is being encouraged.

**Abstract**

 Several grammatical errors need to be addressed throughout the manuscript and abstract. Some examples include;

1. The main aim of this study is to identify the factors **influencing the women** associated with use of LARC and to examine the relationship between knowledge of LARC and its current use.
2. Findings further showed that at both levels of analyses there is a significant relationship (P<0.05 and P=0.00 for binary and multivariable logistic regression, respectively) between knowledge of **LARCs and uses in this study**.
3. Also, the authors should minimize or avoid use of “it” and instead describe it as “LARCs) as was described in the statement; “This means that knowledge of LARC & other socio-demographic variables among women of reproductive age in Nigeria can influence the use of **it.**”

 The authors should specify which “other socio-demographics” to describe in the abstract that are associated with LARC use.

 Revise they conclusion into a total of 2-3 sentences to include the implications of this study on practice, policy and/or research. The authors described the implications in the conclusion of the main manuscript and so should do the same in the abstract

**Main manuscript/document**

**Introduction**

 In the this section, include a citation for the sentence, “Recently, the expansion in choice of contraceptives available has given women the option of adopting the use of Long-acting reversible contraceptives (LARCs) which are implant and intrauterine device contraceptive methods that are highly effective and convenient with an added advantage of being long-lasting,

 require little or no maintenance.

**Results**

 Table 2 is unusually brief with a single row of results. The authors should include the 14.8% prevalence of LARC users (frequency and 95%CI too in order to fit the columns titles already created)

Is the work clearly and accurately presented and does it cite the current literature?

Yes

If applicable, is the statistical analysis and its interpretation appropriate?

Yes

Are all the source data underlying the results available to ensure full reproducibility?

Yes

Is the study design appropriate and is the work technically sound?

Yes

Are the conclusions drawn adequately supported by the results?

Yes

Are sufficient details of methods and analysis provided to allow replication by others?

Yes

Reviewer Expertise:

Reproductive health epidemiology, Global Health and mixed methods research

I confirm that I have read this submission and believe that I have an appropriate level of expertise to confirm that it is of an acceptable scientific standard, however I have significant reservations, as outlined above.

# Response

**Published:** 2020-05-16

**Authors:** Obasanjo Bolarinwa

**Affiliations:** Obafemi Awolowo University, Nigeria

Hello,

 Thanks for this awesome review, please find below my replies bolded.

 Abstract

- **Several grammatical errors need to be addressed throughout the manuscript and abstract. Some**

examples include;

1. The main aim of this study is to identify the factors **influencing the women** associated with use of LARC and to examine the relationship between knowledge of LARC and its current use. - **All grammatical error has been corrected.**
2. Findings further showed that at both levels of analyses there is a significant relationship (P<0.05 and P=0.00 for binary and multivariable logistic regression, respectively) between knowledge of **LARCs and uses in this study**. - **All grammatical error has been corrected.**
3. Also, the authors should minimize or avoid use of “it” and instead describe it as “LARCs) as was described in the statement; “This means that knowledge of LARC & other socio-demographic variables among women of reproductive age in Nigeria can influence the use of it.” – **All grammatical error has been corrected.**

The authors should specify which “other socio-demographics” to describe in the abstract that are associated with LARC use. – **All socio-demographic factors associated has been listed out, there are no “other” socio-demographic factors**.

 Revise they conclusion into a total of 2-3 sentences to include the implications of this study on practice, policy and/or research. The authors described the implications in the conclusion of the main manuscript and so should do the same in the abstract. – **This has been included**

**Main manuscript/document**

 Introduction

 In the this section, include a citation for the sentence, “Recently, the expansion in choice of contraceptives available has given women the option of adopting the use of Long-acting reversible contraceptives available has given women the option of adopting the use of Long-acting reversible contraceptives (LARCs) which are implant and intrauterine device contraceptive methods that are highly effective and convenient with an added advantage of being long-lasting, require little or no maintenance. **– The reference is the second on the reference list. “ *Stoddard A, McNicholas C, Peipert JF: Efficacy and safety of long-acting reversible contraception. Drugs. 2011;71(8):969–980. 21668037 10.2165/11591290-000000000-00000 3662967*” All these references have been listed as 1-4 at the end of the first paragraph, citing “2” at the end of this sentence will distort the referencing style.**

 Results

 Table 2 is unusually brief with a single row of results. The authors should include the 14.8% prevalence of LARC users (frequency and 95%CI too in order to fit the columns titles already created). – **This has been included.**

# Peer review: Reviewer response for version 1

**DOI:** 10.21956/gatesopenres.13998.r27839

**Published:** 2019-09-30

**Authors:** Atem Bethel Ajong, Martin Ndinakie Yakum

**Affiliations:** 1 Medecins Sans Frontieres-Spain (MSF-OCBA), Maiduguri, Nigeria; 2 Department of Obstetrics and Gynaecology, Faculty of Medicine and Biomedical Sciences, University of Yaoundé I, Yaoundé, Cameroon; 3 Department of Biochemistry, University of Dschang, West Region, Cameroon; 4 Department of mother and child health, Kekem District Hospital, West Region, Cameroon

The authors address contraceptive use; a very important pillar in reproductive health. They focus on long acting reversible contraception among current contraceptive users. As already stated by the authors, long acting reversible contraceptives (LARC) are associated with higher contraceptive efficacy (less than 1 % failure rates) and higher rates of contraceptive continuity. Its contribution in shifting the contraceptive method mix to an ideal position in every population is significant and therefore a knowledge of some factors that can influence their uptake is vital.

 The authors evaluate the knowledge of reproductive age women on LARCs and go further to identify some factors associated to LARC use. Globally the manuscript is well drafted with presented results responding only partially to the set objectives.

 One key word in the title of the paper is knowledge but it is unclear if actually the knowledge was evaluated. Having heard of something does not necessary mean you know about it. We must be a little careful; using something gives you the opportunity to know about it. We are of the belief that use can influence knowledge and knowledge can also influence use. For example, you cannot be using IUD and say that you have never heard of it. The key question is what did you know about it before deciding to use? Also, “reproductive age” features as a key word in the title when the study was carried out among contraceptive users. From these analyses, we think the title of the paper should be changed because of its misleading nature. “Factors influencing adoption of LARCs among contraceptive users in Nigeria” is a possible title.

 The write up still requires serious English editing and corrections for a scientific paper (it should probably be edited by a native English speaker). Further statistical analyses are indispensable. Our comments are presented below section wise.

**Abstract**

 Please correct the English thoroughly and make sure the tenses are adapted.

 The very first statement of the abstract is unclear and seems to contradict science unless my reading and understanding is not that of the authors. Reading the statement as it is gives a wrong understanding to the reader. One will think the authors are suggesting a global contraceptive failure rate of 48%. Or do the authors mean “48% of unintended pregnancy that occur in contraceptive user is due to human error”. Please totally rephrase to ease understanding. In addition the references used to state this rate down in your background (ref 5 and 6 are not adapted and have nothing to do with the declarations), please verify and correct.

 Please try to reformulate the objective of the study and in doing so replace the word “examine” which does not seem adapted for this purpose.

 In the method section, please try to include the survey type used to collect this data, include the threshold of significance of the p-value for the identification of the different factors associated with LARCs.

 In the result section, one will want to see at least the mean age and the marital status of the considered population. In addition, you start by presenting the rate of use of traditional methods of contraception which responds to no set objective.

 Major findings like the rate of use of LARCs should be presented with their 95% Confidence intervals.

 Still in your results, you state “findings further revealed…” please avoid using this word revealed.

 Concerning your conclusions, you state “This study concludes….” Please reformulate. You are the one concluding from the results of the study, not the study concluding. In addition, there is serious discordance between your results presented on the abstract and the conclusions. According to your results, LARC use is influenced by the level of knowledge of the women on LARCs but in your conclusion, you state that LARC use is affected by level of education, age of women, …after controlling for confounders which we don’t know yet. Please correct accordingly

**Introduction**

 Please edit and strengthen the English in this section.

 Please reformulate the first sentence of paragraph one. It is not the rising use of contraception that is giving women the ability to choose, but the fact that modern contraception is becoming more and more available and accessible cost to the population.

 Paragraph 2. The authors state “Also LARC methods have the ability to bridge the gap between “typical use” and perfect use” failure rates” Does the use of LARCs reduce or bridge the gap? The word bridge is confusing and makes the reader to wonder on the really message the authors wish to pass out here. Most importantly, the sentence just after the above mentioned sentence (the rest of the whole paragraph) should be reformulated and adequately referenced.

 Paragraph 4. The authors state “despite the level of awareness...”. I wish to remind the authors that the presented awareness rates of the IUD and implants are already low.

 Some background information is lacking. What was the rate of LARC use in Nigeria according the NDHS data? What are some factors that have been identified in Nigeria, and sub-Saharan African or in Africa as a whole to influence the use of LARCs? What about the availability and accessibility of these methods in the study populations? Are they available and trained providers to administer these methods? Please complete this information because it might help understand your findings.

 Your last paragraph should be totally reformulated and made clearer.

**Methods**

 Is there a possibility to cite the methodology used? If yes, it will be better to cite the protocol if it was published.

 What do the authors mean by a weighted sample size? Why a weighted sample size? Detailed information on how the weighted sample size was obtained is indispensable.

 The authors should provide a definition for a current contraceptive user in this study. Because this definition affects the contraceptive method mix. For instance, a condom user who did not engage in sexual activity for a month before the study and therefore did not use a condom; where was she placed? Please a precise definition for a current user is needed.

 The data analysis section is too scanty and difficult to follow. Binary logistic regression generates ORs which are adjusted (AOR) following multiple logistic regression ^1^. What was the threshold of significance for both level of analyses. What criteria did you use to include variables in the multiple logistic regression model? What were the confounders you mentioned in your abstract? What criteria did you used to consider a variable a confounder? Please be more explicit ^2^.

 In addition, as already stated above, analysis of the association of knowledge and use of LARCs is meaningless when the element of knowledge is just “have heard of it”. What do we expect? To say that some women are using and have not heard of it? We really need to know what they know about each method or LARCs as a whole and it will be ideal to try to rather know what they knew about it before initiation of use. Also, the sources of information on the LARCs are good but we have to also know “when”. For example, if you start using something, you can want to know more about it and when you know more by reading through the newspaper, the information you get can rather affect adherence or discontinuation.

**Results**

 Please replace “women currently using any method of contraception or family planning” with women currently using contraception all through your text.

 Please reformulate the sentence presenting the level of education of the participants in paragraph 1. One has the impression that those who are considered in higher education are from the proportion in secondary education, of which it is not the case.

 Please what definition do you give to married here? Do you include only legally married or in this group we have women in consensual unions? Please be precise.

 I think if data on the full contraceptive method mix was available and presented here, it would make this a better manuscript. Please present this data before you focus on LARCs. In the proportion considered to be using LARCs, why was precise data on the IUCD and the implant not included? In addition add a 95% CI to the LARC rate.

 Paragraph 5 from Table 5. Please include how knowledge affects use of LARCs; that is, the direction of association. The last sentence of this paragraph declaring that use of LARCs sole depends on knowledge should be taken off. If this is even true then there is no essence of testing for the other factors presented below. Or are the authors suggesting that all the other factors I see below are confounders?

 The factors presented in this paper were evaluated by simple logistic regression with no confounders defined. The level of education of the participants can affect their wealth index, their number of children, family planning discussion and their likelihood to visit a health worker. Each of the considered factors should logically be controlled for other possible factors. Without this, this section is of no significant importance to the write-up.

**Discussion**

 The whole discussion section has to be retaken. It seems more like a repetition of already presented results than analytically putting findings into context. Please do correct this section.

**Limitations**

 Major limitations associated to this study have not been addressed. Method and design liked limitations have totally been left out. The cross sectional design used to collect the data comes with major limitations allowing us with mere hypotheses than real cause effect relationships. In addition, associations might just have been temporary.

**Future suggestions**

 Your first two sentences are a repetition. Please correct.

**Conclusions**

 The very first sentence of the conclusion has a problem. Please read carefully and correct.

 The second sentence is not correct. No confounders were controlled in the statistical analysis. All the presented factors need to be reviewed after reanalysis.

Is the work clearly and accurately presented and does it cite the current literature?

Partly

If applicable, is the statistical analysis and its interpretation appropriate?

Partly

Are all the source data underlying the results available to ensure full reproducibility?

Yes

Is the study design appropriate and is the work technically sound?

Yes

Are the conclusions drawn adequately supported by the results?

Partly

Are sufficient details of methods and analysis provided to allow replication by others?

Partly

Reviewer Expertise:

Reproductive Health, Public Health and Epidemiology, Clinical Medicine, Clinical Biochemistry.

We confirm that we have read this submission and believe that we have an appropriate level of expertise to confirm that it is of an acceptable scientific standard, however we have significant reservations, as outlined above.

## References

[1] : Model building strategy for logistic regression: purposeful selection. *Ann Transl Med*.2016;4(6) : 10.21037/atm.2016.02.15 111 10.21037/atm.2016.02.15 27127764PMC4828741

[2] : Controlling for continuous confounders in epidemiologic research. *Epidemiology*.1997;8(4) :429-34 9209859

# Response

**Published:** 2020-04-09

**Authors:** Obasanjo Bolarinwa

**Affiliations:** Obafemi Awolowo University, Nigeria

Reply to comments

 The authors evaluate the knowledge of reproductive age women on LARCs and go further to identify some factors associated to LARC use. Globally the manuscript is well drafted with “presented results responding only partially to the set objectives.”

  

1. “One key word in the title of the paper is knowledge but it is unclear if the knowledge was Evaluated”- **We used ever “HEARD” as a proxy to Knowledge**

 

1. Abstract – “The very first statement of the abstract is unclear and seems to contradict science unless my reading and understanding is not that of the authors. Reading the statement as it is gives a wrong understanding to the reader. One will think the authors are suggesting a global contraceptive failure rate of 48%. Or do the authors mean “48% of unintended pregnancy that occur in contraceptive user is due to human error”. Please totally rephrase to ease understanding. In addition the references used to state this rate down in your background (ref 5 and 6 are not adapted and have nothing to do with the declarations), please verify and correct”. Accepted **– Abstract & Intro – Reference added.**

 

1. “Please try to reformulate the objective of the study and in doing so replace the word “examine” which does not seem adapted for this purpose”. **Accepted – Changed the word examine in the objective to identify and re-arranged.**

 

1. “In the method section, please try to include the survey type used to collect this data, include the threshold of significance of the p-value for the identification of the different factors associated with LARCs” . **Accepted- In the method section survey type included.**

 

1. “In the result section, one will want to see at least the mean age and the marital status of the considered population. In addition, you start by presenting the rate of use of traditional methods of contraception which responds to no set objective. – **Table 1 presented the mean age and marital status**

 

1. Major findings like the rate of use of LARCs should be presented with their 95% Confidence intervals. – **Done**

**Use of LARC with their 95% Confidence intervals.**

**Variable **​​​​​​ **Std. Err.** **95% CI**

**​​​​​​​**

 LARC use ​​​0.1320 – 0.1638

1. “Still in your results, you state “findings further revealed…” please avoid using this word revealed. – **Acknowledged.**

 

1. “Concerning your conclusions, you state “This study concludes….” Please reformulate. You are the one concluding from the results of the study, not the study concluding. In addition, there is serious discordance between your results presented on the abstract and the conclusions. According to your results, LARC use is influenced by the level of knowledge of the women on LARCs but in your conclusion, you state that LARC use is affected by level of education, age of women, …after controlling for confounders which we don’t know yet. Please correct accordingly” – **Corrected **

 

1. Introduction - Please edit and strengthen the English in this section. - **Acknowledged.**

 

1. Please reformulate the first sentence of paragraph one. It is not the rising use of contraception that is giving women the ability to choose, but the fact that modern contraception is becoming more and more available and accessible cost to the population. - **Acknowledged.**

 

1. Paragraph 2. The authors state “Also LARC methods have the ability to bridge the gap between “typical use” and perfect use” failure rates” Does the use of LARCs reduce or bridge the gap? The word bridge is confusing and makes the reader to wonder on the really message the authors wish to pass out here. Most importantly, the sentence just after the above mentioned sentence (the rest of the whole paragraph) should be reformulated and adequately referenced. - **Acknowledged.**

 

1. Paragraph 4. The authors state “despite the level of awareness...”. I wish to remind the authors that the presented awareness rates of the IUD and implants are already low. - **Acknowledged.**

 

1. Some background information is lacking. What was the rate of LARC use in Nigeria according the NDHS data? What are some factors that have been identified in Nigeria, and sub-Saharan African or in Africa as a whole to influence the use of LARCs? What about the availability and accessibility of these methods in the study populations? Are they available and trained providers to administer these methods? Please complete this information because it might help understand your findings. - **Acknowledged.**

 

1. Your last paragraph should be totally reformulated and made clearer.- **Acknowledged.**

 

1. Methods - Is there a possibility to cite the methodology used? If yes, it will be better to cite the protocol if it was published.- **Done**

 

1. What do the authors mean by a weighted sample size? Why a weighted sample size? Detailed information on how the weighted sample size was obtained is indispensable. – **Acknowledged and corrected**

 

1. The authors should provide a definition for a current contraceptive user in this study. Because this definition affects the contraceptive method mix. For instance, a condom user who did not engage in sexual activity for a month before the study and therefore did not use a condom; where was she placed? Please a precise definition for a current user is needed. **– Done**

 

**women ages 15–49 who are using (or whose partners are using) any contraceptive method at the time of the survey**

  

1. The data analysis section is too scanty and difficult to follow. Binary logistic regression generates ORs which are adjusted (AOR) following multiple logistic regression . What was the threshold of significance for both level of analyses. What criteria did you use to include variables in the multiple logistic regression model? What were the confounders you mentioned in your abstract? What criteria did you used to consider a variable a confounder? Please be more explicit .In addition, as already stated above, analysis of the association of knowledge and use of LARCs is meaningless when the element of knowledge is just “have heard of it”. What do we expect? To say that some women are using and have not heard of it? We really need to know what they know about each method or LARCs as a whole and it will be ideal to try to rather know what they knew about it before initiation of use. Also, the sources of information on the LARCs are good but we have to also know“when”. For example, if you start using something, you can want to know more about it and when you

know more by reading through the newspaper, the information you get can rather affect adherence or discontinuation. – **Done**

**The variable were added to logistic regression based on literature review and association at chi-square level. Knowledge of LARC and Use of LARC has been analyzed (Check Table 5 )**

  

1. Results - Please replace “women currently using any method of contraception or family planning” with women currently using contraception all through your text. - **Acknowledged.**

 

1. Please reformulate the sentence presenting the level of education of the participants in paragraph 1. One has the impression that those who are considered in higher education are from the proportion in secondary education, of which it is not the case.- **Done **

 

  

1. Please what definition do you give to married here? Do you include only legally married or in this group we have women in consensual unions? Please be precise. I think if data on the full contraceptive method mix was available and presented here, it would make this a better manuscript. Please present this data before you focus on LARCs. In the proportion considered to be using LARCs, why was precise data on the IUCD and the implant not included? In addition add a 95% CI to the LARC rate.- **Done **

 

 Method Mix of current user

**Married includes married women and those who are living with a man**

  

1. Paragraph 5 from Table 5. Please include how knowledge affects use of LARCs; that is, the direction of association. The last sentence of this paragraph declaring that use of LARCs sole depends on knowledge should be taken off. If this is even true then there is no essence of testing for the other factors presented below. Or are the authors suggesting that all the other factors I see below are confounders?- **Done**

 

1. The factors presented in this paper were evaluated by simple logistic regression with no confounders defined. The level of education of the participants can affect their wealth index, their number of children, family planning discussion and their likelihood to visit a health worker. Each of the considered factors should logically be controlled for other possible factors. Without this, this section is of no significant importance to the write-up. - **Corrected**

 

1. Discussion - The whole discussion section has to be retaken. It seems more like a repetition of already presented results than analytically putting findings into context. Please do correct this section. - **Corrected**

 

1. Limitations - Major limitations associated to this study have not been addressed. Method and design liked limitations have totally been left out. The cross sectional design used to collect the data comes with major limitations allowing us with mere hypotheses than real cause effect relationships. In addition, associations might just have been temporary. **Done**

 

1. Future suggestions - Your first two sentences are a repetition. Please correct. **Corrected**

 

1. Conclusions - The very first sentence of the conclusion has a problem. Please read carefully and correct. The second sentence is not correct. No confounders were controlled in the statistical analysis. All the presented factors need to be reviewed after reanalysis. **Done**
